# Sickness absence diagnoses among abstainers, low‐risk drinkers and at‐risk drinkers: consideration of the U‐shaped association between alcohol use and sickness absence in four cohort studies

**DOI:** 10.1111/add.14249

**Published:** 2018-06-05

**Authors:** Jenni Ervasti, Mika Kivimäki, Jenny Head, Marcel Goldberg, Guillaume Airagnes, Jaana Pentti, Tuula Oksanen, Paula Salo, Sakari Suominen, Markus Jokela, Jussi Vahtera, Marie Zins, Marianna Virtanen

**Affiliations:** ^1^ Finnish Institute of Occupational Health Helsinki Finland; ^2^ Department of Epidemiology and Public Health University College London UK; ^3^ Clinicum University of Helsinki Helsinki Finland; ^4^ Population‐based Cohorts Unit French National Institute of Health and Medical Research (INSERM) Villejuif France; ^5^ Research Unit 1168 Aging and Chronic Diseases—Epidemiological and Public Health Approaches French National Institute of Health and Medical Research (INSERM) Villejuif France; ^6^ Université Paris Descartes Sorbonne Paris Cité Paris France; ^7^ Department of Psychiatry and Addictology AP‐HP, Hôpitaux Universitaires Paris Ouest Paris France; ^8^ Department of Psychology University of Turku Finland; ^9^ University of Turku, Finland and University of Skövde Sweden; ^10^ Medicum University of Helsinki Helsinki Finland; ^11^ University of Turku and Turku University Hospital Turku Finland; ^12^ Department of Public Health and Caring Sciences University of Uppsala Uppsala Sweden

**Keywords:** Abstaining, at‐risk drinking, longitudinal, multi‐cohort, pooled data, sick leave

## Abstract

**Aims:**

To estimate differences in the strength and shape of associations between alcohol use and diagnosis‐specific sickness absence.

**Design:**

A multi‐cohort study. Participants (*n* = 47 520) responded to a survey on alcohol use at two time‐points, and were linked to records of sickness absence. Diagnosis‐specific sickness absence was followed for 4–7 years from the latter survey.

**Setting and participants:**

From Finland, we had population cohort survey data from 1998 and 2003 and employee cohort survey data from 2000–02 and 2004. From France and the United Kingdom, we had employee cohort survey data from 1993 and 1997, and 1985–88 and 1991–94, respectively.

**Measurements:**

We used standard questionnaires to assess alcohol intake categorized into 0, 1–11 and > 11 units per week in women and 0, 1–34 and > 34 units per week in men. We identified groups with stable and changing alcohol use over time. We linked participants to records from sickness absence registers. Diagnoses of sickness absence were coded according to the International Classification of Diseases. Estimates were adjusted for sex, age, socio‐economic status, smoking and body mass index.

**Findings:**

Women who reported drinking 1–11 units and men who reported drinking 1–34 units of alcohol per week in both surveys were the reference group. Compared with them, women and men who reported no alcohol use in either survey had a higher risk of sickness absence due to mental disorders [rate ratio = 1.51, 95% confidence interval (CI) = 1.22–1.88], musculoskeletal disorders (1.22, 95% CI = 1.06–1.41), diseases of the digestive system (1.35, 95% CI = 1.02–1.77) and diseases of the respiratory system (1.49, 95% CI = 1.29–1.72). Women who reported alcohol consumption of > 11 weekly units and men who reported alcohol consumption of > 34 units per week in both surveys were at increased risk of absence due to injury or poisoning (1.44, 95% CI = 1.13–1.83).

**Conclusions:**

In Finland, France and the United Kingdom, people who report not drinking any alcohol on two occasions several years apart appear to have a higher prevalence of sickness absence from work with chronic somatic and mental illness diagnoses than those drinking below a risk threshold of 11 units per week for women and 34 units per week for men. Persistent at‐risk drinking in Finland, France and the United Kingdom appears to be related to increased absence due to injury or poisoning.

## Introduction

Previous studies have shown that both abstainers and at‐risk drinkers are at an increased risk of sickness absence compared with low‐risk drinkers 1, 2, 3, 4, 5. As former at‐risk drinkers also have a higher risk of sickness absence than low‐risk drinkers 6, part of the excess sickness absence in abstainers may be attributable to health selection, i.e. at‐risk drinkers may reduce their drinking due to health problems, and thus the higher risk of sickness absence among abstainers is due at least partly to prior alcohol use.

Differences in social insurance systems, policies for sickness absence and cultural differences might affect the association between alcohol use and sickness absence 7, 8, 9, 10. To obtain generalizable, rather than particular evidence, in our previous study we used data from three countries (Finland, the United Kingdom and France), and demonstrated a U‐shaped association between alcohol use and sickness absence among men 11. In that study, both men and women abstaining from alcohol for 4–6 years had a higher risk of all‐cause sickness absence compared to those who had been moderate drinkers during the same time. To date, however, it is still unclear which specific diagnoses are associated with the excess risk of sickness absence among abstainers, and whether they correspond to those observed among at‐risk drinkers.

The first aim of the current study was to estimate differences in diagnosis‐specific sickness absence diagnosis between abstainers, former, persistent and new at‐risk and low‐risk drinkers. We focused on mental disorders, musculoskeletal disorders, diseases of the circulatory system, digestive disease and respiratory diseases, as well as external causes, as these diagnostic groups have been found to underlie alcohol‐related sickness absence 12, 13. In addition, we studied whether the alcohol use–sickness absence associations differed between men and women.

## Methods

### Design

In this prospective multi‐cohort study, we used individual‐level data from four well‐characterized cohort studies: (a) a population‐based sample of Finnish working‐age adults participating in the Health and Social Support (HeSSup) study, Finland 14, 15; (b) the Whitehall II study of British civil servants 16; (c) the employees of the national gas and electricity company participating in the GAZEL study, France 17; and (d) the municipal employees of the Finnish Public Sector (FPS) study, Finland 1. In all these studies, alcohol use was assessed twice, and diagnosis‐specific sickness absences were followed for 4–7 years after the latter survey via linkage to electronic health records. Ethical approval was obtained from Turku University Central Hospital Ethics committee for the HeSSup study, from the University College London Medical School committee on the ethics of human research for the Whitehall II study, from the Inserm Ethics committee for GAZEL and from the Ethics committee of the Hospital District of Helsinki and Uusimaa for FPS.

From all four cohorts, we included respondents who were alive, not retired before the start of the follow‐up and had data on all studied variables from the surveys that were included in this study design. The eligible population in each study comprised the respondents of a baseline and follow‐up questionnaire survey. In the HeSSup study, the survey years were 1998 and 2003 (*n* = 10 667), in the GAZEL study 1993 and 1997 (*n* = 8107), in the Whitehall II study phases 1 (1985–8) and 3 (1991–94) (*n* = 3730) and in the FPS 2000–02 and 2004 (*n* = 25 016). The attrition rates between the two measurement points were acceptable: 24% were lost to follow‐up in HeSSup, 7% in GAZEL, 13% in Whitehall II and 34% in FPS.

The follow‐up time (time at‐risk for sickness absence) in all studies was until disability or old‐age pension, death or end of follow‐up, whichever came first. Time at‐risk for sickness absence was defined with person‐years in the labour force. In the Whitehall II cohort, we were able exclude possible periods of unemployment, parental leave or other transient episodes of not working from time at risk. In the GAZEL, HeSSup and FPS cohorts we were unable to exclude these episodes of not working.

### Measurement of alcohol use

Alcohol use was assessed by questions on average weekly consumption. One drink/alcohol unit was estimated as 12 g of alcohol (EUR unit) except in Whitehall, where a unit was 8 g (UK unit). Alcohol intake was categorized into ‘no alcohol use’, ‘moderate use’ (a maximum of 140 g or 1–11 EUR units or 1–17 UK units for women and 280 g or 1–23 EUR units or 1–34 UK units for men per week), and ‘heavy use’ (> 140 g or > 11 EUR units or > 17 UK units for women and > 280 g or > 23 EUR units or > 34 UK units for men per week). The cut‐points of at‐risk drinking were based on Finnish Current Care Guidelines 18.

Alcohol use was measured twice (two survey responses). Based on these two measurements, we classified the respondents as ‘abstainers’ (no alcohol use in either survey), ‘low‐risk’ (moderate use reported in both surveys), ‘former at‐risk’ (heavy use reported at baseline survey, but no or moderate use in the follow‐up survey), ‘persistent at‐risk’ (heavy use reported in both surveys) and ‘new at‐risk’ (heavy use at follow‐up survey only). Those who changed between abstinence and moderate use between the two time‐points were omitted from the study (pooled *n* = 4924, 10%). This was conducted to ensure the ‘cleanliness’ reference group, i.e. low‐risk drinkers. Changing between abstinence and moderate use could be a sign of health problems intervening with our study design. Classification of alcohol use was consistent with our previous study 11.

### Ascertainment of diagnosis‐specific sickness absence

Sickness absence was measured as number of sickness absence days per follow‐up year. In HeSSup and FPS, register information on the dates of sickness absence exceeding 9 days was retrieved from the Social Insurance Institution of Finland. These were followed‐up from 1 January 2004 to 31 December 2010 in HeSSup and from 1 January 2005 to 31 December 2011 in FPS. In Whitehall II, information on all days of sickness absence was from the Civil Service (employer) records for those employees who gave consent to monitor their sickness absence for a follow‐up period from phase 3 until the end of 1998. In GAZEL, the information on annual days of sickness absence was obtained from the employer's records for a follow‐up period from 1 anuary 1998 to 31 December 2004.

Diagnoses of sickness absence were coded according to International Classification of Diseases (ICD‐10) in HeSSup, GAZEL and FPS. We used the following diagnosis groups: codes F00‐F99 for mental and behavioural disorder; I00–I99 for diseases of the circulatory system; J00–J99 for diseases of the respiratory system; K00–K93 for diseases of the digestive system; M00–M99 for diseases of the musculoskeletal system and connective tissue; and S00–T98 for injury, poisoning and certain other consequences of external causes. In HeSSup and FPS, the data included all absence episodes lasting for at least 10 days, from the date that illness began (the first days of absence form work) until the sickness absence benefit ended. In GAZEL, a medical certificate is required from day 1, and the data included all episodes of sickness absence irrespective of length. However, the cause of sickness absence was missing for 50% of absences of fewer than 7 days, 17% of those of 8–28 days and 3% of absences of more than 28 days 12.

In Whitehall II, a medical certificate was required for absences longer than 7 calendar days. The coding was based on ICD‐8 classification, which was converted to a smaller number of disease categories using the morbidity coding system of the Royal College of General Practitioners (RCGP), as described elsewhere in detail 19, 20. We used codes 5 (psychoses), 40 (neurosis) and 41 (neurosis ill‐defined) to define sickness absence due to mental and behavioural disorder‐related sickness absence; codes 9 (cardiovascular diseases), 10 (cerebrovascular diseases) and 11 (peripheral vascular diseases) to define sickness absence due to diseases of the circulatory system; code 12 (diseases of the respiratory system); code 13 (diseases of the digestive system); code 17 (diseases of the musculoskeletal system and connective tissue); and code 23 (injury and poisoning). Thus, our outcome measures best capture sickness absence due to chronic long‐term illness rather than transient/short‐term illness, such as respiratory infections, headaches or migraine.

### Covariates

Covariates, measured at T2, were socio‐economic status (SES), age, sex, smoking and body mass index (BMI). SES was based on occupational class except for HeSSup, where information on occupational class was unavailable, and SES was based on vocational education. In FPS and GAZEL, SES was based on register data and in HeSSup and Whitehall, it was based on self‐reports. High SES included administrators, managers, experts and specialists, and in HeSSup, those with university/polytechnic education. Intermediate SES included skilled non‐manual occupations, such as office work, customer service, sales work and hospital nurses, and in HeSSup, those with college‐level education. Low SES included manual workers, such as construction workers, manufacturing, transportation (FPS, GAZEL), clerical and office support work (in Whitehall II) and those with vocational school, vocational course, apprenticeship training or no vocational education (HeSSup).

Age was treated as a continuous variable in the analyses, except for sickness absence due to musculoskeletal diagnoses, where we observed a curvilinear association with age among women. There, we used age as categorized into < 40, < 50 and ≥ 50 years. Smoking was self‐reported in all studies, and was dichotomized into current smoker or non‐smoker (including never and ex‐smokers). BMI (= weight in kg divided by height in m^2^) was self‐reported in HeSSup, GAZEL and FPS. In the Whitehall II study, BMI was derived from measures taken at clinical examinations. BMI was categorized as less than 18.5 (underweight), 18.5–25 (normal weight), 25–29 (overweight) and 30 or more (obesity).

### Statistical analysis

First, we used a two‐step meta‐analysis 21. In the first step, we performed study‐specific analyses and used negative binomial regression analysis to examine the rate ratios (RR) with their 95% confidence intervals (CI) of sickness absence for no alcohol use, former at‐risk drinking, persistent at‐risk drinking and new at‐risk drinking compared to persistent low‐risk drinking. We adjusted for sex, age, SES, smoking and BMI. The study‐specific results were analysed using SAS version 9.4. In the second step, study‐specific RR estimates were pooled in fixed‐effects meta‐analysis with Stata version 14 software. We examined heterogeneity between the estimates using the *I* ^2^ statistic. As a sensitivity analysis, we pooled study specific estimates in random‐effects meta‐analysis.

As a supplementary analysis, we also performed a single‐step meta‐analysis, where the data from the four cohorts were pooled together and analysed with negative binomial regression analysis. Here, study cohort as well as sex, age, SES, smoking and BMI were treated as fixed‐effects, i.e. covariates. Interaction term ‘sex × alcohol use’ were tested for each outcome adjusted for covariates and found non‐significant (*P* > 0.05). As a sensitivity analysis to this single‐step meta‐analysis, we used the generalized estimating equations (GEE) framework (with negative binomial distribution). To account for clustering of participants within cohorts, we added ‘cohort’ as cluster factor (independent correlation structure).

## Results

The risk factors for sickness absence (older age, lower SES, smoking and obesity) in each cohort by alcohol use are described in Table 1. In HeSSup, persistent at‐risk drinkers had the highest average age. In the three other cohorts, the differences in mean age by alcohol use were smaller. In all cohorts, the prevalence of low SES was highest among abstainers. Smoking was most common among at‐risk drinkers (either former, persistent or new). Obesity was most common among persistent or new at‐risk drinkers in all cohorts, except for HeSSup, where obesity was most prevalent among abstainers (Table 1).

**Table 1:** Characteristics of participants in the four cohorts by alcohol use.

HeSSup	Persistent abstainers n = 989 (9%)	Persistent low‐risk n = 8360 (78%)	Former at‐risk n = 440 (4%)	Persistent at‐risk n = 356 (3%)	New at‐risk n = 522 (5%)
Mean age (SD)	43 (11)	42 (11)	40 (12)	46 (9)	42 (10)
Women, %	72	56	70	65	68
High SES, %	23	33	28	29	27
Intermediate SES, %	30	32	32	33	35
Low SES, %	48	35	40	39	38
Smoking, %	14	19	33	39	39
BMI < 18.5, %	1.9	1.1	1.6	0.6	1.3
BMI 18.5–24.9, %	51	53	51	46	50
BMI 25–30, %	32	35	35	41	36
BMI > 30, %	14	11	13	12	13

Whitehall II	Persistent abstainers n = 453 (12%)	Persistent low‐risk n = 2810 (75%)	Former at‐risk n = 159 (4%)	Persistent at‐risk n = 182 (5%)	New at‐risk n = 126 (3%)
Mean age (SD)	50 (6)	48 (6)	48 (5)	47 (5)	47 (5)
Women, %	49	22	26	25	25
High SES, %	13	46	47	40	50
Intermediate SES, %	48	44	44	55	44
Low SES, %	39	10	9	5	6
Smoking, %	14	13	23	30	20
BMI < 18.5, %	2.0	0.8	1.9	0.6	0.8
BMI 18.5–24.9, %	51	54	45	47	42
BMI 25–30, %	33	38	43	43	40
BMI > 30, %	14	8	10	10	17

GAZEL	Persistent abstainers n = 570 (7%)	Persistent low‐risk n = 5578 (69%)	Former at‐risk n = 508 (6%)	Persistent at‐risk n = 958 (12%)	New at‐risk n = 493 (6%)
Mean age (SD)	50 (3)	51 (3)	51 (3)	52 (3)	51 (3)
Women, %	57	24	19	16	22
High SES, %	24	42	42	38	42
Intermediate SES, %	59	49	50	51	47
Low SES, %	17	9	7	11	11
Smoking, %	14	16	21	31	22
BMI < 18.5, %	2.1	0.7	0.2	0.5	0.8
BMI 18.5–24.9, %	59	47	42	42	44
BMI 25–30, %	29	45	49	48	45
BMI > 30, %	10	8	9	9	10

FPS	Persistent abstainers n = 2532 (10%)	Persistent low‐risk n = 18 786 (75%)	Former at‐risk n = 1021 (4%)	Persistent at‐risk n = 1384 (6%)	New at‐risk n = 1293 (5%)
Mean age (SD)	49 (8)	48 (8)	47 (8)	50 (7)	47 (8)
Women, %	89	80	80	77	81
High SES, %	47	58	58	66	59
Intermediate SES, %	34	28	27	22	27
Low SES, %	19	14	15	13	14
Smoking, %	10	14	26	25	25
BMI < 18.5, %	0.6	0.9	0.7	0.9	0.5
BMI 18.5–24.9, %	53	53	49	44	51
BMI 25–30, %	31	34	35	39	36
BMI >30, %	15	12	15	17	13

FPS = Finnish Public Sector study; HeSSup = Health and Social Support Study; SD = standard deviation; SES = socio‐economic status; BMI = body mass index.

We tested the ‘sex × alcohol use’ interaction with both pooled data (adjusted for covariates) and with meta‐regression. All *P*‐values for sex × alcohol use interaction were > 0.05. Thus, we present the results in the whole data set adjusting for sex.

In Supporting information, Table S1, we present the observed (i.e. unadjusted) mean days of diagnosis‐specific sickness absence days per person‐year by alcohol use in each cohort. For most diagnoses, mean absence days were highest either among persistent abstainers or among persistent at‐risk drinkers, corresponding to a U‐shaped relationship.

### RR of diagnosis‐specific sickness absence: two‐step meta‐analysis

**Figure 1:**
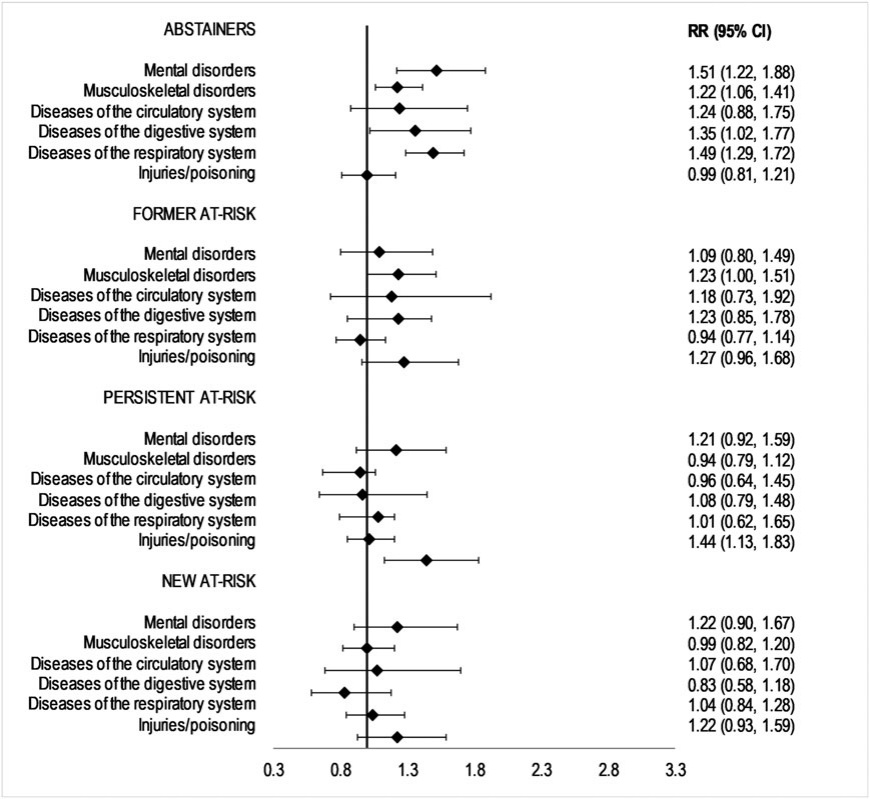
Pooled rate ratios (95% confidence intervals) for the association between alcohol use and sickness absence (n = 47 520). Adjusted for age, socio‐economic status, smoking and body mass index. Abstainers, former, persistent and new at‐risk drinkers are compared to low‐risk drinkers

Figure 1 shows the pooled estimates of risk of sickness absence according to alcohol use. Compared to low‐risk drinkers (the reference group), there was a higher risk for sickness absence due to mental disorders for abstainers. People abstaining from alcohol also had a higher risk of sickness absence due to musculoskeletal disorders, diseases of the digestive system and disease of the respiratory system. Former at‐risk drinkers had a higher risk of sickness absence due to musculoskeletal disorder. Persistent at‐risk drinkers had a higher risk of sickness absence due to external causes.

The study‐specific estimates (+between‐study heterogeneity values) and pooled random‐effects estimates are shown in Supporting information, Figs S1–S6. Significant between‐study heterogeneity was observed in sickness absence due to diseases of the respiratory system (*I* ^2^ = 75%, *P* = 0.007) among abstainers (Supporting information, Fig. S5). All other statistically significant associations were without significant heterogeneity. The estimates from random‐effects meta‐analysis corresponded to those from fixed‐effects meta‐analysis, except for wider confidence intervals. The pooled random‐effects estimate for abstainers on sickness absence due to diseases of the respiratory system was not significant (RR = 1.30, 95% CI = 0.91, 1.86).

### RR of diagnosis‐specific sickness absence: single‐step meta‐analysis

Table 2 shows the observed (unadjusted) mean days sickness absence per person‐years and the estimates for the association between alcohol use and diagnosis‐specific sickness absence from the pooled data. These results corresponded to those from two‐step meta‐analysis. Abstaining from alcohol was associated with higher risk of sickness absence due to mental, musculoskeletal, digestive and respiratory diagnoses. Former at‐risk drinking was associated with higher risk of musculoskeletal disorder‐related sickness absence. Persistent at‐risk drinking was associated with higher risk of sickness absence related to external causes.

**Table 2:** Adjusted* rate ratios (95% CIs) for the association between alcohol use and diagnosis of sickness absence; pooled data (n = 47 520).

	Abstainers n = 4730 (10%)		Low‐risk n = 36 733 (75%)	Former at‐risk n = 2211 (4%)		Persistent at‐risk n = 2984 (6%)		New at‐risk n = 2539 (5%)	
Mental									
Mean days/person‐years	2.2		1.4	1.8		2.1		2.0	
RR (95% CI)	1.58	(1.27, 1.96)	1	1.02	(0.75, 1.39)	1.24	(0.94, 1.62)	1.20	(0.90, 1.59)
Musculoskeletal									
Mean days/person‐years	4.2		3.1	3.3		3.2		3.5	
RR (95% CI)	1.26	(1.09, 1.46)	1	1.23	(1.00, 1.50)	0.99	(0.83, 1.18)	0.99	(0.82, 1.20)
Circulatory									
Mean days/person‐years	0.6		0.4	0.6		0.5		0.4	
RR (95% CI)	1.39	(0.99, 1.97)	1	1.22	(0.75, 1.96)	1.14	(0.75, 1.74)	1.29	(0.82, 2.02)
Digestive									
Mean days/person‐years	0.3		0.2	0.4		0.3		0.2	
RR (95% CI)	1.38	(1.04, 1.82)	1	1.37	(0.92, 2.02)	1.26	(0.89, 1.79)	0.87	(0.60, 1.25)
Respiratory									
Mean days/person‐years	0.6		0.4	0.4		0.5		0.3	
RR (95% CI)	1.35	(1.13, 1.62)	1	1.01	(0.79, 1.29)	0.94	(0.76, 1.17)	0.97	(0.77, 1.23)
Injury/poisoning									
Mean days/person‐years	1.1		1.0	1.1		1.5		1.5	
RR (95% CI)	1.02	(0.83, 1.26)	1	1.22	(0.92, 1.62)	1.32	(1.03, 1.70)	1.40	(1.07, 1.83)

* Adjusted for age, socio‐economic status, smoking, body mass index and cohort. CI = confidence interval; RR = rate ratio.

Sensitivity analyses with cohort as repeated subject yielded additional statistically significant (*P* < 0.05) estimates. In these analyses, abstaining was associated also with sickness absence due to diseases of the circulatory system. Former at‐risk drinking was associated additionally with higher risk of sickness absence due to diseases of the circulatory and digestive system. Sickness absence due to external causes was higher among new at‐risk drinkers, in addition to persistent at‐risk drinkers (Supporting information, Table S2).

Finally, we tested whether combining all at‐risk drinkers (regardless of change in drinking) to a single group would affect the results. The results corresponded to earlier results: at‐risk drinking was associated with sickness absence due to external causes (RR = 1.32, 95% CI = 1.12, 1.55, Supporting information, Table S3).

## Discussion

This pooled analysis of four cohorts from three countries provides new evidence about diseases underlying the U‐shaped association between alcohol use and sickness absence. Compared to low‐risk drinkers, abstainers had a higher risk of sickness absence due to mental disorders, musculoskeletal disorders, diseases of the respiratory system and diseases of the digestive system. Former at‐risk drinkers had a higher risk of musculoskeletal disorder‐related sickness absence. Persistent at‐risk drinkers had a higher risk of sickness absence due to external causes (i.e. injuries or poisoning) compared to low‐risk drinkers.

Our findings demonstrate that the curvilinear association, i.e. the higher risk of sickness absence among both abstainers and at‐risk drinkers, relates to a different set of diagnosis of sickness absence for the two risk groups. Several issues other than the effect of not using alcohol may explain the higher absence rates due to both somatic and mental diagnoses in abstainers. For example, psychotrophic medication and diseases of the digestive system often prevent alcohol use. Thus, the association between abstaining and increased risk of sickness absence could be due in part to health selection; that is, groups with increased disease burden, such as former heavy drinkers and people abstaining due to health reasons, may have been selected into the group of abstainers. In our data, at‐risk drinkers in the first survey but not in the second survey, i.e. former at‐risk drinkers, had an elevated risk of sickness absence due to musculoskeletal disorders compared to low‐risk drinkers. Moreover, former at‐risk drinkers may have an elevated risk of short‐term sickness absence, but our data captured chronic, long‐term illnesses most effectively.

At the other end of alcohol use, the link between heavy drinking and poisoning is well established 22, 23, 24, and at least two meta‐analyses have found higher alcohol intake to be associated with increased risk of injury 25, 26. Our study supported these associations: persistent at‐risk drinkers had more sickness absence due to external causes than low‐risk drinkers. People classified into persistent at‐risk group may have more excessive drinking episodes, which more probably result in injuries or even alcohol poisoning. In a previous study, binge drinking was related to injury‐related absenteeism 13.

Another explanation may be the healthy worker effect, if participants to whom at‐risk drinking causes health problems are selected out from the labour market; that is, if they retire early or become unemployed. Then, the adverse health effects are not seen in absence from work due to illness.

### Strengths and limitations

Our study has several strengths, including a prospective design, measuring alcohol use twice over time allowing us to assess change in drinking behaviours and reliable register‐based sickness absence data with diagnosis codes. Moreover, we could control for many life‐style‐related confounding factors.

There were also limitations, including the inability to control for life‐time abstinence. However, in a previous study with FPS data, both life‐time and current abstinence were linked with higher risk of sickness absence 1. In Finland, the diagnoses of sickness absence episodes are registered only for absences lasting for more than 9 days. Thus, information on short‐term episodes were available for only GAZEL and Whitehall II cohorts. This affected probably mainly sickness absence due to respiratory diseases, where mild influenza, common cold or other acute upper respiratory infections not usually causing work disability for 10 days are not recorded for FPS and HeSSup. Consequently, the relative weight of Whitehall II and GAZEL are much larger in these respiratory diagnoses. However, approximately 50% of data on sickness absence diagnosis was missing for short‐term absences in GAZEL data, which might have caused bias to our results. Limitations also include self‐report data on alcohol use, which may cause measurement error if at‐risk drinkers were included in low‐risk group. This would suggest that our estimates may be underestimates of the true effect. However, we have shown previously that using a different (UK‐recommended) cut‐off point for at‐risk drinking produces similar associations to sickness absence 11.

Moreover, in HeSSup, FPS and GAZEL, we were unable to identify transient absence from the labour force (such as parental leaves, sabbatical, studying or unemployment) from the at‐risk time. However, in Finland, people receiving parental, study, unemployment or sabbatical benefits are entitled to sickness benefit if they become ill (after the 10‐day reimbursement period), and thus they were technically at‐risk of sickness absence even if they were not working. In the French GAZEL cohort, the participants were ageing (mean age 51 years) at the beginning of the follow‐up. Thus, during the 7‐year follow‐up, 80% of them retired (at an average age of 55 years). There were hardly any parental leaves in this cohort.

Finally, differences between sickness absence certification schemes between countries can cause residual confounding. However, we had very little significant between‐study heterogeneity, which increases the strength of evidence.

## Conclusion

People abstaining from alcohol had a higher risk of sickness absence due to chronic mental and somatic disorders compared to low‐risk drinkers, and persistent at‐risk drinkers had more sickness absence due to external causes compared to persistently low‐risk drinkers. The U‐shaped alcohol use–sickness absence association seems thus to comprise differential diagnostic patterns at both ends of alcohol use continuum. Our results can help occupational health care and facilitate early interventions/auditing for at‐risk alcohol use when accumulating sickness absence due to external causes are observed.

## Declaration of interests

None.

## Supporting information


**Table S1** Observed diagnosis‐specific sickness absence days by alcohol use in each cohort. The highest mean on each row is shown in bold type.
**Table S2** Adjusted* rate ratios (95% confidence intervals) for the association between alcohol use and diagnosis of sickness absence. Pooled data (n = 47 520).
**Table S3** Adjusted* rate ratios (95% confidence intervals) for the association between alcohol use and diagnosis of sickness absence. Pooled data (n = 47 520).
**Figure S1** Rate ratios (95% confidence intervals) for the association between alcohol use and sickness absence due to mental disorders in each study cohort (n = 47 520). Abstainers, former, persistent and new at‐risk drinkers are compared to low‐risk drinkers. Adjusted for age, socio‐economic status, smoking and body mass index. I–V = fixed‐effects model; D + L = random‐effects model.
**Figure S2** Rate ratios (95% confidence intervals) for the association between alcohol use and sickness absence due to musculoskeletal disorders in each study cohort (n = 47 520). Abstainers, former, persistent and new at‐risk drinkers are compared to low‐risk drinkers. Adjusted for age, socio‐economic status, smoking and body mass index. I–V = fixed‐effects model; D + L = random‐effects model.
**Figure S3** Rate ratios (95% confidence intervals) for the association between alcohol use and sickness absence due to diseases of the circulatory system in each study cohort (n = 47 520). Abstainers, former, persistent and new at‐risk drinkers are compared to low‐risk drinkers. Adjusted for age, socio‐economic status, smoking and body mass index. I–V = fixed‐effects model; D + L = random‐effects model.
**Figure S4** Rate ratios (95% confidence intervals) for the association between alcohol use and sickness absence due to diseases of the digestive system in each study cohort (n = 47 520). Abstainers, former, persistent and new at‐risk drinkers are compared to low‐risk drinkers. Adjusted for age, socio‐economic status, smoking, and body mass index. I–V = fixed‐effects model; D + L = random‐effects model.
**Figure S5** Rate ratios (95% confidence intervals) for the association between alcohol use and sickness absence due to diseases of the respiratory system in each study cohort (n = 47 520). Abstainers, former, persistent and new at‐risk drinkers are compared to low‐risk drinkers. Adjusted for age, socio‐economic status, smoking and body mass index. I–V = fixed‐effects model; D + L = random‐effects model.
**Figure S6** Rate ratios (95% confidence intervals) the association between alcohol use and sickness absence due to injury/poisoning in each study cohort (n = 47 520). Abstainers, former, persistent and new at‐risk drinkers are compared to low‐risk drinkers. Adjusted for age, socio‐economic status, smoking and body mass index. I–V = fixed‐effects model; D + L = random‐effects model.
