# Supplementary material for: Sickness absence diagnoses among abstainers, low‐risk drinkers and at‐risk drinkers: consideration of the U‐shaped association between alcohol use and sickness absence in four cohort studies
Source: Addiction. 2018 Jun 5;113(9):1633–42. doi: 10.1111/add.14249 (PMC6099368; doi:10.1111/add.14249)
Supplement: Supplementary file 1 — Table S1 Observed diagnosis‐specific sickness absence days by alcohol use in each cohort. The highest mean on each row is shown in bold type. Table S2 Adjusted* rate ratios (95% confidence intervals) for the association between alcohol use and diagnosis of sickness absence. Pooled data (n = 47 520). Table S3 Adjusted* rate ratios (95% confidence intervals) for the association between alcohol use and diagnosis of sickness absence. Pooled data (n = 47 520). Figure S1 Rate ratios (95% confidence intervals) for the association between alcohol use and sickness absence due to mental disorders in each study cohort (n = 47 520). Abstainers, former, persistent and new at‐risk drinkers are compared to low‐risk drinkers. Adjusted for age, socio‐economic status, smoking and body mass index. I–V = fixed‐effects model; D + L = random‐effects model. Figure S2 Rate ratios (95% confidence intervals) for the association between alcohol use and sickness absence due to musculoskeletal disorders in each study cohort (n = 47 520). Abstainers, former, persistent and new at‐risk drinkers are compared to low‐risk drinkers. Adjusted for age, socio‐economic status, smoking and body mass index. I–V = fixed‐effects model; D + L = random‐effects model. Figure S3 Rate ratios (95% confidence intervals) for the association between alcohol use and sickness absence due to diseases of the circulatory system in each study cohort (n = 47 520). Abstainers, former, persistent and new at‐risk drinkers are compared to low‐risk drinkers. Adjusted for age, socio‐economic status, smoking and body mass index. I–V = fixed‐effects model; D + L = random‐effects model. Figure S4 Rate ratios (95% confidence intervals) for the association between alcohol use and sickness absence due to diseases of the digestive system in each study cohort (n = 47 520). Abstainers, former, persistent and new at‐risk drinkers are compared to low‐risk drinkers. Adjusted for age, socio‐economic status, smoking, and body mass index. I–V = [file ADD-113-1633-s001.docx]

**Supplementary Table 1.** Observed diagnosis-specific sickness absence days by alcohol use in each cohort. The highest mean on each row is bolded.

| **HeSSup** |  |  |  |  |  |
| --- | --- | --- | --- | --- | --- |
| Mean days of sickness absence per person-years | Persistent abstainers n=989 (9%) | Persistent low-risk n=8360 (78%) | Former at-risk n=440 (4%) | Persistent at-risk n=356 (3%) | New at-risk n=522 (5%) |
| Mental | 1.79 | 1.31 | 1.02 | **2.47** | 1.91 |
| Circulatory diseases | 0.33 | 0.30 | **0.50** | 0.45 | 0.35 |
| Musculoskeletal | 3.16 | 2.36 | 2.85 | **3.36** | 2.58 |
| Digestive | 0.24 | 0.19 | 0.21 | **0.39** | 0.15 |
| Respiratory | **0.29** | 0.20 | **0.29** | 0.18 | 0.20 |
| Injury or poisoning | 0.75 | 0.84 | 0.95 | **1.24** | 1.07 |
| **Whitehall II** |  |  |  |  |  |
| Mean days of sickness absence per person-years | Persistent abstainers n=453 (12%) | Persistent low-risk n=2810 (75%) | Former at-risk n=159 (4%) | Persistent at-risk n=182 (5%) | New at-risk n=126 (3%) |
| Mental | 1.65 | 0.99 | 2.05 | **3.21** | 1.07 |
| Circulatory diseases | **0.74** | 0.17 | 0.003 | 0.49 | 0.21 |
| Musculoskeletal | **2.03** | 0.79 | 0.27 | 0.66 | 0.28 |
| Digestive | 0.66 | 0.19 | **0.42** | 0.13 | 0.18 |
| Respiratory | **3.17** | 1.47 | 1.33 | 2.02 | 1.18 |
| Injury or poisoning | **1.39** | 0.47 | 1.05 | 0.73 | 0.12 |
| **GAZEL** |  |  |  |  |  |
| Mean days of sickness absence per person-years | Persistent abstainers n=570 (7%) | Persistent low-risk n=5578 (69%) | Former at-risk n=508 (6%) | Persistent at-risk n=958 (12%) | New at-risk n=493 (6%) |
| Mental | **2.14** | 1.25 | 1.29 | 1.15 | 1.01 |
| Circulatory diseases | 0.45 | 0.64 | 0.74 | **0.96** | 0.76 |
| Musculoskeletal | **2.59** | 1.39 | 2.02 | 1.96 | 1.75 |
| Digestive | 0.44 | 0.43 | 0.45 | **0.58** | 0.38 |
| Respiratory | 0.29 | 0.41 | 0.47 | 0.41 | **0.52** |
| Injury or poisoning | 1.13 | 1.24 | 0.98 | 1.74 | **1.88** |
| **FPS** |  |  |  |  |  |
| Mean days of sickness absence per person-years | Persistent abstainers n=2532 (10%) | Persistent low-risk n=18786 (75%) | Former at-risk n=1021 (4%) | Persistent at-risk n=1384 (6%) | New at-risk n=1293 (5%) |
| Mental | **2.45** | 1.67 | 2.14 | 2.28 | 2.33 |
| Circulatory diseases | 0.65 | 0.50 | **0.76** | 0.41 | 0.33 |
| Musculoskeletal | **3.47** | 1.65 | 2.15 | 2.20 | 2.20 |
| Digestive | 0.45 | **0.51** | 0.41 | **0.51** | 0.39 |
| Respiratory | **0.63** | 0.52 | 0.54 | 0.51 | 0.57 |
| Injury or poisoning | 1.82 | 1.29 | 1.32 | 1.82 | **2.31** |

**Supplementary Table 2.** Adjusted* rate ratios (95% Cis) for the association between alcohol use and diagnosis of sickness absence. Pooled data (n=47 520).

|  | Abstainers n=4730 (10%) | | Low-risk n=36 733 (75%) | | Former at-risk n=2211 (4%) | | Persistent at-risk n=2984 (6%) | | New at-risk n=2539 (5%) | |
| --- | --- | --- | --- | --- | --- | --- | --- | --- | --- | --- |
|  | RR | 95% CI | RR | 95% CI | RR | 95% CI | RR | 95% CI | RR | 95% CI |
| Mental | 1.60 | 1.46-1.76 | 1 |  | 1.02 | 0.96-1.09 | 1.21 | 0.91-1.60 | 1.26 | 1.08-1.47 |
| Musculoskeletal | 1.16 | 1.07-1.25 | 1 |  | 1.21 | 1.07-1.36 | 0.94 | 0.73-1.20 | 1.04 | 0.95-1.13 |
| Circulatory | 1.24 | 1.08-1.44 | 1 |  | 1.34 | 1.16-1.54 | 1.16 | 0.58-2.30 | 1.40 | 0.95-2.05 |
| Digestive | 1.31 | 1.13-1.52 | 1 |  | 1.46 | 1.12-1.90 | 1.34 | 0.98-1.82 | 0.90 | 0.87-0.94 |
| Respiratory | 1.45 | 1.11-1.90 | 1 |  | 0.96 | 0.83-1.11 | 0.96 | 0.86-1.07 | 1.05 | 0.70-1.57 |
| Injury/poisoning | 0.97 | 0.88-1.07 | 1 |  | 1.18 | 0.99-1.41 | 1.42 | 1.18-1.71 | 1.53 | 1.30-1.81 |

* Adjusted for age, socioeconomic status, smoking, and body mass index. GEE modelling (negative binomial distribution): repeated subject = cohort; type of correlation structure = independent.

**Supplementary Table 3.** Adjusted* rate ratios (95% Cis) for the association between alcohol use and diagnosis of sickness absence. Pooled data (n=47 520).

|  | Abstainers n=4730 (10%) |  | Low-risk n=36 733 (75%) | At-risk** n=6057 (15%) |  |
| --- | --- | --- | --- | --- | --- |
|  | RR | 95% CI | RR (Referent) | RR | 95% CI |
| Mental | 1.58 | 1.27-1.96 | 1 | 1.16 | 0.97-1.39 |
| Musculoskeletal | 1.24 | 1.07-1.20 | 1 | 1.07 | 0.95-1.20 |
| Circulatory | 1.39 | 0.99-1.97 | 1 | 1.21 | 0.92-1.60 |
| Digestive | 1.38 | 1.04-1.82 | 1 | 1.16 | 0.93-1.45 |
| Respiratory | 1.35 | 1.13-1.62 | 1 | 0.97 | 0.84-1.12 |
| Injury or poisoning | 1.03 | 0.83-1.26 | 1 | 1.32 | 1.12-1.55 |

* Adjusted for age, socioeconomic status, smoking, and body mass index, and cohort

** At-risk drinking either at T1, T2, or both


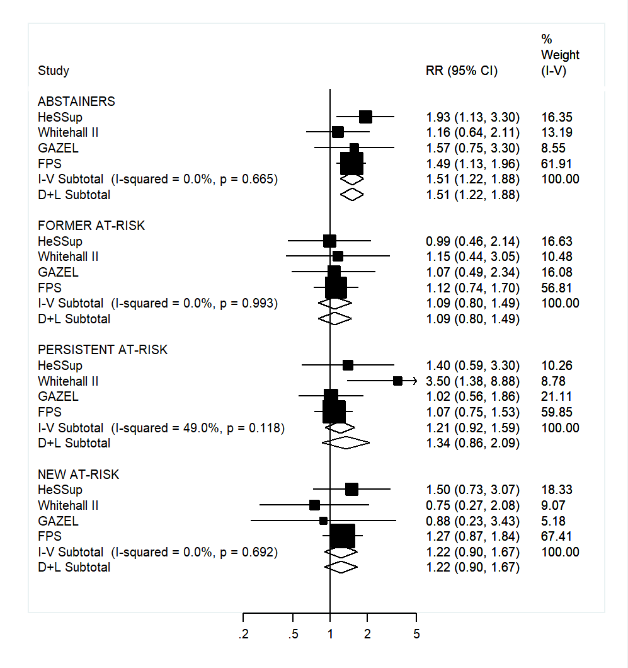


**Supplementary Figure 1**. Rate ratios (95% CIs) for the association between alcohol use and sickness absence due to mental disorders in each study cohort **(n=47 520)**. Abstainers, former, persistent, and new at-risk drinkers are compared to low-risk drinkers. Adjusted for age, socioeconomic status, smoking, and body mass index. I-V = Fixed effects model; D+L = Random effects model.


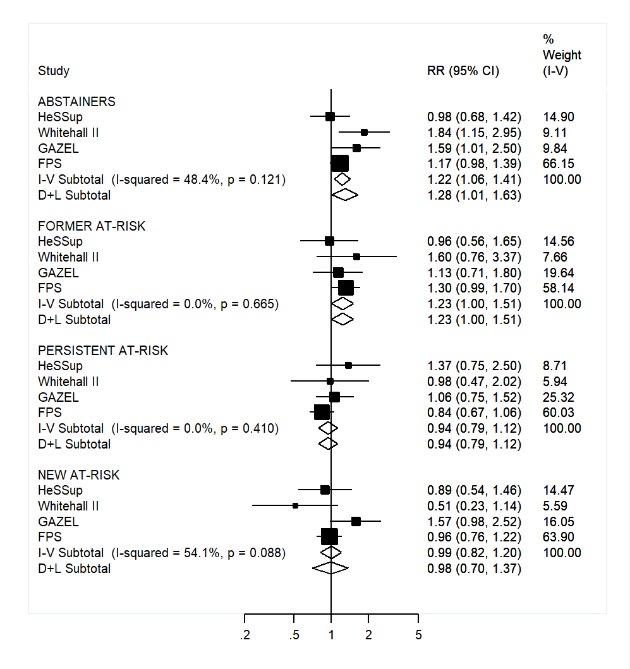


**Supplementary Figure 2**. Rate ratios (95% CIs) for the association between alcohol use and sickness absence due to musculoskeletal disorders in each study cohort **(n=47 520)**. Abstainers, former, persistent, and new at-risk drinkers are compared to low-risk drinkers. Adjusted for age, socioeconomic status, smoking, and body mass index. I-V = Fixed effects model; D+L = Random effects model.


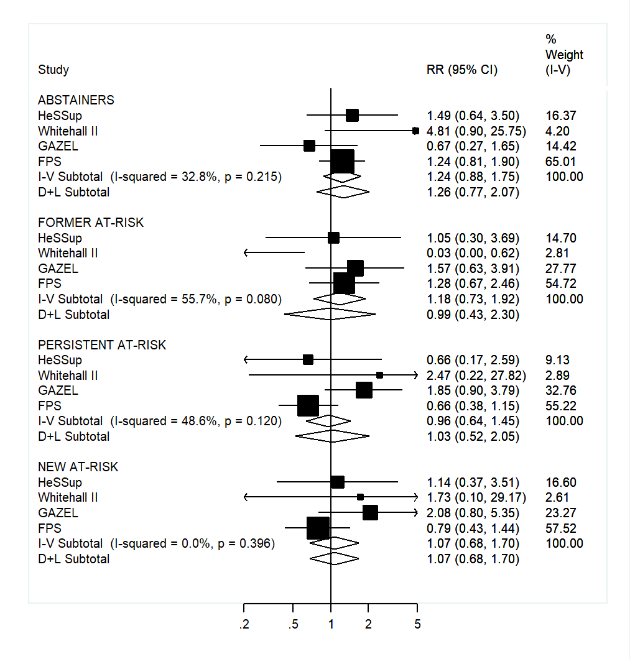


**Supplementary Figure 3**. Rate ratios (95% CIs) for the association between alcohol use and sickness absence due to diseases of the circulatory system in each study cohort **(n=47 520)**. Abstainers, former, persistent, and new at-risk drinkers are compared to low-risk drinkers. Adjusted for age, socioeconomic status, smoking, and body mass index. I-V = Fixed effects model; D+L = Random effects model.


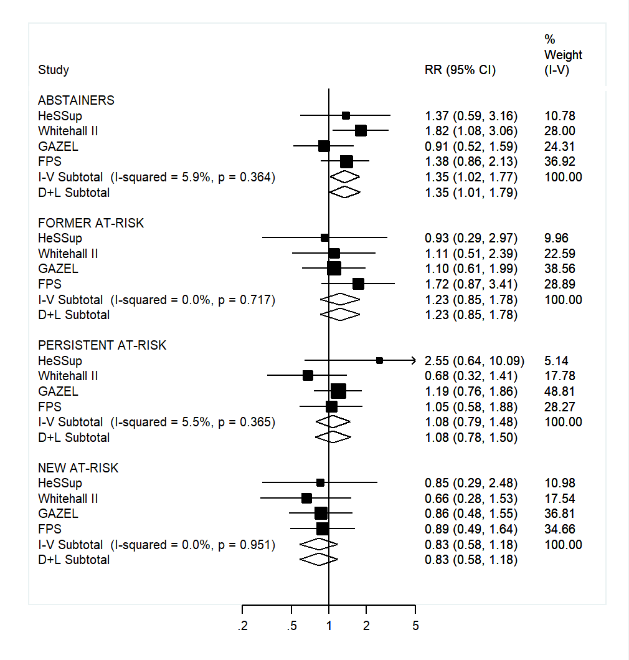


**Supplementary Figure 4**. Rate ratios (95% CIs) for the association between alcohol use and sickness absence due to diseases of the digestive system in each study cohort **(n=47 520)**. Abstainers, former, persistent, and new at-risk drinkers are compared to low-risk drinkers. Adjusted for age, socioeconomic status, smoking, and body mass index. I-V = Fixed effects model; D+L = Random effects model.


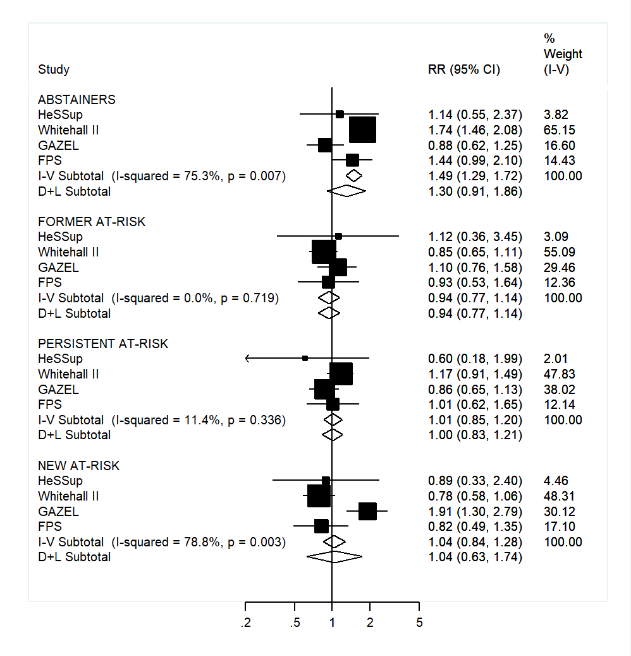


**Supplementary Figure 5**. Rate ratios (95% CIs) for the association between alcohol use and sickness absence due to diseases of the respiratory system in each study cohort **(n=47 520)**. Abstainers, former, persistent, and new at-risk drinkers are compared to low-risk drinkers. Adjusted for age, socioeconomic status, smoking, and body mass index. I-V = Fixed effects model; D+L = Random effects model.


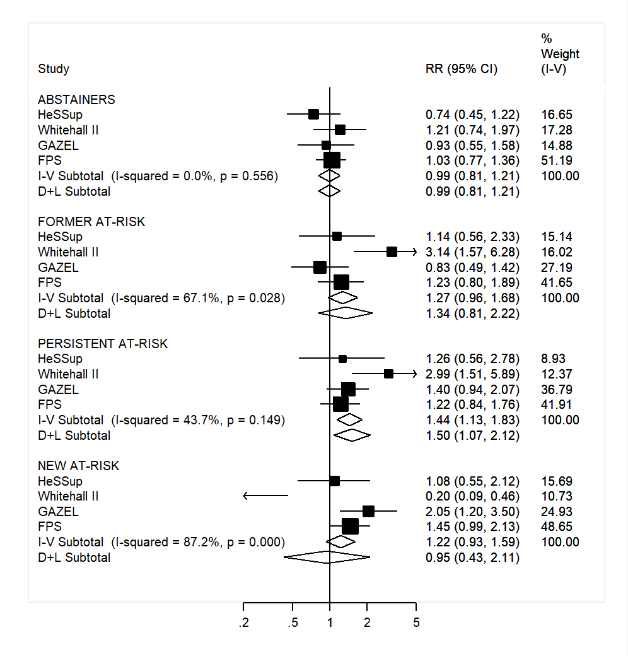


**Supplementary Figure 6**. Rate ratios (95% CIs) for the association between alcohol use and sickness absence due to injury/poisoning in each study cohort **(n=47 520)**. Abstainers, former, persistent, and new at-risk drinkers are compared to low-risk drinkers. Adjusted for age, socioeconomic status, smoking, and body mass index. I-V = Fixed effects model; D+L = Random effects model.
